# Editorial: Advances in Oil Crops Research—Classical and New Approaches to Achieve Sustainable Productivity

**DOI:** 10.3389/fpls.2019.00791

**Published:** 2019-06-18

**Authors:** Dragana Miladinović, Johann Vollmann, Leire Molinero-Ruiz, Mariela Torres

**Affiliations:** ^1^Sunflower Department, Institute of Field and Vegetable Crops, Novi Sad, Serbia; ^2^Department of Crop Sciences, University of Natural Resources and Life Sciences Vienna, Vienna, Austria; ^3^Departamento de Protección de Cultivos, Instituto de Agricultura Sostenible, Córdoba, Spain; ^4^Estación Experimental Agropecuaria San Juan, Instituto Nacional de Tecnología Agropecuaria, Buenos Aires, Argentina

**Keywords:** oil crops, breeding, quality, production, diversity

The world production of main oil crops is steadily increasing, mainly due to population growth and increased use of oil crops in bio-fuel production and in edible vegetable oils. From the perspective of sowing area in the world, oil crops are only preceded by cereals in importance. Edible or industrial oils are extracted from seeds, fruits or mesocarp, and nuts of both annual and perennial species. Oil can be obtained from about 40 different crops, but soybean, sunflower, olive tree, and rapeseed have a major importance in the total world trade. The purpose of the Research Topic “Advances in Oil Crops Research—Classical and New Approaches to Achieve Sustainable Productivity” is to provide the reader compiled information of the latest research results about different aspects of oil crops.

This research topic incorporates 23 publications including 19 research papers, three review articles, and one perspective.

## Diversity Characterization

Germplasm collections are very important for conservation of oil crop diversity. In olive tree, only few collections have been fully genotyped and characterized. Mousavi et al. screened previously uncharacterized olive trees from the olive collection of Perugia University with standard simple sequence repeats (SSR) and new expressed sequence tag (EST)-SSR markers. They found the new OLea Expressed Sequence Tags (OLEST) SSR markers to be as effective as other commonly used markers. OLEST-SSR markers are very useful to create a common database from worldwide collections of olive cultivars.

Jatropha has been recognized as one of the best candidates for future biodiesel production. Attempts for commercial cultivation of the species in Africa and Asia have failed due to low productivity. Thus, better yielding commercial cultivars must be developed on the basis of an increased knowledge on the species genetic diversity. Li et al. analyzed the genetic diversity of 246 Jatropha accessions collected in Africa, Asia, and Mesoamerica by means of SSR and retrotransposon-based insertion polymorphism markers. The authors identified that two accessions from Veracruz (Mesoamerica) were the source of all Jatropha of Africa and Asia, and hypothesized that human selection caused low Jatropha productivity. They have also suggested that Jatropha of Africa and Asia can be improved through the implementation of breeding strategies.

## Environment and Agronomy

Olive is a crop very well-adapted to temperature and precipitation regimes typical of regions in the Mediterranean Basin. The increasing international demand for olive oil has led to the expansion of olive cultivation in new production regions where environmental conditions differ from those of the Mediterranean Basin and there is little information about olive adaptation to these new environments. Torres et al. reviewed the scientific literature on olive cultivation in non-Mediterranean environments, with focus on chilling requirements for flowering, water requirements, and irrigation management, as well as environmental effects on fruit oil content and quality. Their information could be useful to determine whether environmental conditions in new growing regions are appropriate for achieving sustainable olive and oil productions. Several simulation models of the olive crop have been formulated so far, but none of them is capable to account for the impacts of environmental conditions and management practices on growth and productivity in the absence of nutrient deficiencies, diseases, and pests. López-Bernal et al. described OliveCan, a process-oriented model formulated using previous models by the group, that enables the assessment of combined effects of management operations, soil traits, and weather over crop performance both under unstressed and water deficit conditions.

Functional-structural plant modeling (FSPM) is a dynamic method for prediction of plant growth under varying environmental conditions. As the temperature is a primary factor affecting growth and development of rapeseed, Tian et al. tested three different temperature treatments by using a FSPM of seedlings with a growth function used for leaf extension and biomass accumulation implemented by combining measurements with literature data.

## Biotic Stresses

Effective, economically viable, and environmentally friendly methods for pest control are needed in the framework of sustainable agriculture in Europe. These novel methods are particularly important when dealing with insect pests that have developed insecticide resistance, such as olive fruit fly. Yousef et al. tested the efficacy of soil treatment with the entomopathogenic fungus *Metarhizium brunneum* for controlling the olive fly and found that, during spring, the density of pest population emerging from the soil was reduced by up to 70%. They also studied the retention of conidia as a function of soil type and rain amount.

Several secondary metabolites can be produced by capitate glandular trichomes (CGT). Some of these secondary metabolites provide durable resistance to insect pests. The specialist pest sunflower moth is combated by host resistance based on CGT. Gao et al. have identified two major QTLs controlling CGT density in sunflower florets. They constructed a genetic linkage map from genotyping-by-sequencing data using SNP markers. The obtained results will advance the understanding of CGT-mediated resistance to insect pests in sunflower. The authors also provide a resource for marker-assisted selection for insect resistance in this oil crop species.

Anthracnose is an important disease that causes fruit rot and branch dieback of olive trees. The appearance of symptoms and their evolution in time are highly dependent on cultivar susceptibility and environmental conditions. Moral et al. looked for potential sources of resistance to *Colletotrichum acutatum*, causal agent of anthracnose, in the World Olive Germplasm Bank located in Córdoba (Spain). The authors also described the original methodology that could be also used in the evaluation of olive cultivar responses to other aerial fungi affecting leaves and fruit.

*Verticillium dahliae* is a soil-borne fungus that causes wilt and leaf mottle in sunflower. The incidence of this disease of sunflower has increased in recent years consequently affecting sunflower oil production. Martin-Sanz et al. described the population structure of *V. dahliae* affecting sunflowers in Europe. According to the results of genetic, molecular, and pathogenic analyses of particular traits of the isolates, they identified two groups. One was diverse and included *V. dahliae* isolates from Eastern Europe. The other was highly homogeneous and clustered isolates from Western Europe. The authors recommend taking into account the existence of these two groups of *V. dahliae* when looking for sources of resistance to this disease in European environments.

*Orobanche cumana* L. is one of the main limiting factors in sunflower production. Infection of this parasitic weed occurs early after sowing and affects host physiology from that moment and during underground parasite stages. Ortiz-Bustos et al. used the blue-green fluorescence (BGF) emission and thermal imaging of leaves for the detection of the infection of sunflower by *O. cumana* during underground parasite development. Lower BGF emissions and higher temperatures were detected in leaves of infected plants as compared to those of healthy plants, indicating that BGF imaging and thermography could be used for fast and non-destructive screening of lines of sunflower from breeding programs for resistance to this parasitic weed.

## Product Quality

The high genetic diversity of wild olives can be used for introduction of some important agronomic traits into cultivated varieties. However, some of the traits introduced from wild relatives can have negative effects on some other desirable traits such as the composition of virgin olive oil (VOO). As VOO from olive cultivars is highly appreciated for its fatty acid composition, León et al. compared the fatty acid profiles of VOO from wild olives and olive cultivars. They found that the use of wild germplasm in olive breeding programs does not have a negative impact on fatty acid composition, tocopherol content, and tocopherol and phytosterol profiles if the selection for these traits is conducted from early generations of crossings.

Phenolic compounds that are present in olive oil have a strong antioxidant activity and are one of the factors responsible for health benefits associated with VOO consumption. However, phenolic composition is not a common selection criterion in olive breeding programs. A simplified procedure for phenolic profiling of the olive fruits avoiding the previous step of oil extraction was developed by Pérez et al. When applying it in the detection of phenolic content variability in different olive genotypes, a high genotypic variance of fruit phenol content was found in the tested genotypes. The authors concluded that the observed high genotypic variance together with the simplified method for fruit phenol evaluation can be useful in olive tree breeding for improved phenolic profile of the oil. Velázquez-Palmero et al. studied genes and enzymes responsible for the phenolic composition of VOO, such as β-glucosidases. They found that olive *GLU* gene expression is cultivar-dependent and regulated by temperature, light, and water regime, as well as its transcription in olive fruit spatially and temporally regulated. Their study is a further step in elucidating the factors involved in biosynthesis of the major phenolic compounds in VOO. It can also help to develop molecular markers for the selection of cultivars with improved oil quality characteristics.

## Plant Nutrition

Nitrogen fertilization allows farmers to improve plant productivity. However, it has to be carefully managed to avoid harmful environmental impacts due to nitrogen loss. Increasing nitrogen use efficiency (NUE), or the amount of N fertilizer taken up and used by the crop, is vital to solve the conflict between productivity and the protection of the environment. The NUE of rapeseed is low, although it has a high uptake capacity for inorganic N. The development of cultivars with improved NUE is a major goal in rapeseed breeding. A collection of 30 elite rapeseed varieties registered between 1989 and 2014 was tested by Stahl et al. under two different fertilization regimes in a 2-year experiment in 10 different environments for changes in seed yield and seed quality traits. Their data revealed that genetic improvement in combination with reduced N fertilizer inputs has a tremendous potential to increase NUE of rapeseed.

Using a transcriptome approach, Safavi-Rizi et al. found an opposing effect of N depletion on gene regulation: some genes were up- or downregulated in N-depleted lower canopy leaves, contrarily to their regulation in upper leaves under ample N supply. Also, some genes were expressed as associated to senescence in rapeseed, but not in *Arabidopsis*. They hypothesized that some of these genes may have rapeseed-specific functions in nitrogen remobilization during N-deficiency induced leaf senescence. Some genes also seem to contribute to differences in senescence and N resources mobilization in upper and lower canopy leaves.

## Plant Growth and Development

Production of olive trees relies on the successful achievement of sexual reproduction. Due to self-incompatibility in the species, successful fertilization is highly favored by the presence of pollen grains from a different cultivar. Zafra et al. studied the reproductive biology of the olive through identification of key gene products involved in pollen and pistil physiology. Aiming at elucidating the biological processes occurring during the courtship, pollen grain germination, and fertilization in olive, they constructed SSH libraries using pistil and pollen that allowed the identification of transcripts with important roles in stigma physiology.

In the MADS-Box gene family, MADS intervening keratin-like and MIKC-type are found only in plants. They encode transcription factors that have major roles in plant growth and developmental processes. However, a comprehensive analysis of this gene family in *G. hirsutum*, concerning characterization and functions, has not yet been reported. Ren et al. analyzed MIKC-type MADS genes in the tetraploid cotton, which is the most widely cultivated cotton species. A total of 110 *GhMIKC* genes were identified, located in the genome, and phylogenetically classified into 13 subfamilies. Since most of them were highly expressed in floral organs, the study provides useful information of the involvement of *GhMIKCs*' regulation in cotton flowering.

## Molecular Tools for Breeding

In their review, Dimitrijević and Horn elaborated present and past achievements in sunflower breeding and biotechnology, as well as the future perspectives of using modern molecular tools in this research area. They emphasized that the key for further successes in sunflower breeding is in integrative approaches to the genetics of complex quantitative traits and physiological and biochemical mechanisms involved, a challenge that must be faced with new high-throughput technologies in combination with new genomic-based breeding strategies.

Genomic selection (GS) models can predict characters performance after being trained on phenotypic and genotypic information for those characters, and their most important advantage is that they do not have to include all hybrid parents. Mangin et al. compared the accuracy of sunflower oil content prediction of commonly used general combining ability (GCA) and genomic predictions. Their results show that GS provides more accurate predictions compared to the classical predictions based on GCA with at least one parent is untested or not well-characterized, thus increasing breeding speediness and efficiency.

The lack of identified marker-trait associations is a major limitation toward development of successful marker-assisted breeding programs in safflower. Ambreen et al. tested a safflower panel (CartAP) comprising of 124 accessions for its suitability for association mapping. They have detected several marker-trait associations, either in a majority or in some environments. The results of this study will facilitate wider use of marker-assisted breeding in safflower, as well as identification of genetic determinants of trait variability.

Soybean flowering time and maturity are controlled by *E* genes whose different allelic combinations determine soybean adaptation to a certain latitude. Miladinović et al. have described the first attempt to assess adaptation of soybean genotypes developed at Institute of Field and Vegetable Crops, Novi Sad, Serbia based on *E* gene variation, as well as to comparatively assess *E* gene variation in North-American, Chinese and European genotypes. As *e1-as/e2/E3/E4* was the most common genotype and was present in the top performing genotypes, this specific allele combination was proposed as optimal for the environments of Central-Eastern Europe.

Despite its economic and nutritional importance, genetic improvement of sesame lags behind that of the other oil crops. In their review, Dossa et al. described the evolution of research in sesame genetics and highlighted the recent advances in the—omics area. They have also discussed the future prospects for genetic improvement and better expansion of sesame. In another study, Dossa et al. developed SisatBase, an user-friendly database providing free access to SSRs data of sesame. It is as well an integrated platform for functional analyses of this oil crop.

## Conclusion

The paraphrased last sentence from the review of Dimitrijević and Horn is a perfect conclusion message for the topic and a recommendation for future work on oil crops:

“…Only a combined effort of the oil crop research community can make oil crops more competitive to other crops. The new high-throughput technologies combined with new genomic-based breeding strategies give us the opportunity, as never before, to understand and mine genetic variation and to use it for improvement of oil crops…”.

## Author Contributions

All authors listed have made a substantial, direct and intellectual contribution to the work, and approved it for publication.

### Conflict of Interest Statement

The authors declare that the research was conducted in the absence of any commercial or financial relationships that could be construed as a potential conflict of interest.

